# Hypnotic prescription trends and patterns for the treatment of insomnia in Japan: analysis of a nationwide Japanese claims database

**DOI:** 10.1186/s12888-023-04683-2

**Published:** 2023-04-20

**Authors:** Shoki Okuda, Zaina P. Qureshi, Yukiko Yanagida, Chie Ito, Yuji Homma, Shigeru Tokita

**Affiliations:** 1 Medical Affairs, MSD K.K., Tokyo, Japan; 2 Center for Observational and Real-World Evidence (CORE), Merck & Co., Inc., Rahway, NJ USA; 3 JMDC Inc., Tokyo, Japan

**Keywords:** Insomnia, Hypnotics, Prescriptions, Japan, Orexin receptor antagonist, Benzodiazepine, z-drugs, Melatonin receptor antagonist, Claims database

## Abstract

**Background:**

There is limited consensus regarding the optimal treatment of insomnia. The recent introduction of orexin receptor antagonists (ORA) has increased the available treatment options. However, the prescribing patterns of hypnotics in Japan have not been comprehensively assessed. We performed analyses of a claims database to investigate the real-world use of hypnotics for treating insomnia in Japan.

**Methods:**

Data were retrieved for outpatients (aged ≥ 20 to < 75 years old) prescribed ≥ 1 hypnotic for a diagnosis of insomnia between April 1^st^, 2009 and March 31^st^, 2020, with ≥ 12 months of continuous enrolment in the JMDC Claims Database. Patients were classified as new or long-term users of hypnotics. Long-term use was defined as prescription of the same mechanism of action (MOA) for ≥ 180 days. We analyzed the trends (2010–2019) and patterns (2018–2019) in hypnotics prescriptions.

**Results:**

We analyzed data for 130,177 new and 91,215 long-term users (2010–2019). Most new users were prescribed one MOA per year (97.1%–97.9%). In 2010, GABA_A_-receptor agonists (benzodiazepines [BZD] or z-drugs) were prescribed to 94.0% of new users. Prescriptions for BZD declined from 54.8% of patients in 2010 to 30.5% in 2019, whereas z-drug prescriptions remained stable (~ 40%). Prescriptions for melatonin receptor agonist increased slightly (3.2% to 6.3%). Prescriptions for ORA increased over this time from 0% to 20.2%. Prescriptions for BZD alone among long-term users decreased steadily from 68.3% in 2010 to 49.7% in 2019. Prescriptions for ORA were lower among long-term users (0% in 2010, 4.3% in 2019) relative to new users. Using data from 2018–2019, multiple (≥ 2) MOAs were prescribed to a higher proportion of long-term (18.2%) than new (2.8%) users. The distribution of MOAs according to psychiatric comorbidities, segmented by age or sex, revealed higher proportions of BZD prescriptions in elderly (new and long-term users) and male (new users) patients in all comorbidity segments.

**Conclusion:**

Prescriptions for hypnotics among new and long-term users in Japan showed distinct patterns and trends. Further understanding of the treatment options for insomnia with accumulating evidence for the risk–benefit balance might be beneficial for physicians prescribing hypnotics in real-world settings.

**Supplementary Information:**

The online version contains supplementary material available at 10.1186/s12888-023-04683-2.

## Background

Insomnia is a serious condition characterized by nocturnal and diurnal symptoms that may persist for a long time and places a significant burden on society [1]. Insomnia can be managed with either pharmacological therapy, non-pharmacological therapy, or a combination of both [2]. Cognitive behavioral therapy for insomnia (CBTi), a non-pharmacological therapy, is recommended as a first-line treatment in several countries [2–5] and is often preferred by patients [6]. However, it is not yet recommended as first-line treatment and is not covered by health insurance in Japan [7]. Pharmacological therapy has long been the mainstay treatment in Japan [7], and the Japanese Society of Sleep Research suggests that CBTi should be considered for patients who cannot be treated with a regular dose of hypnotics [7].

Historically, the GABA_A_-receptor agonists (GABA_A_-RAs) such as benzodiazepines (BZDs) and non-benzodiazepines (z-drugs) have been the predominantly prescribed hypnotics in Japan. According to a United Nations International Narcotics Control Board report, BZDs were more frequently used as sedative hypnotics in Japan than in other countries for the period 2011–2013 [8]. However, BZDs are associated with physical dependence during long-term use and there are some safety concerns, such as increased risks of fall and fractures among elderly individuals [9–11]. The Japanese Pharmaceuticals and Medical Devices Agency also issued an alert in 2017 warning of dependence associated with GABA_A_-RAs and to limit their long-term/chronic use [12].

The introduction of two novel classes of hypnotics, melatonin receptor agonists (MRA) with the launch of ramelteon in 2010, and orexin receptor antagonists (ORA) with the launch of suvorexant in 2014 and lemborexant in 2020, has evolved the treatment landscape for insomnia in Japan [13–15]. Therefore, data regarding the trends or current patterns in prescriptions of hypnotics for insomnia in a real-world setting in Japan are of interest. Although some studies have examined the prescribing trends and patterns of hypnotics in Japan using claims databases [16–19], the data are still limited and the majority of prior studies focused on BZD (and z-drugs) and did not evaluate MRA or ORA. Therefore, more up-to-date information regarding the trends and patterns of hypnotics prescriptions, including the use of MRA and ORA, is needed to provide insight into how hypnotics are prescribed in Japan.

Our objectives in this study were to investigate the prescribing patterns and trends of hypnotics for insomnia patients using a claims database in Japan. We used data collected between April 2010 and March 2020, which enabled us to assess the past and present landscapes regarding the use of hypnotics, including MRA and ORA. This also allowed us to gain insight into how the introduction of these new classes of hypnotics influenced the prescribing patterns or trends in patients with insomnia. We also investigated the hypnotics prescribing patterns and trends in new users and long-term users of hypnotics.

## Methods

### Data source and study design

For this retrospective observational cohort study, we used data from the JMDC Claims Database, the largest health insurance claims database in Japan [20]. The JMDC Claims Database collects claims data for individuals who belong to health insurance providers for company employees and their family as part of the Japanese union-managed health insurance system (Health Insurance Association). The JMDC Claims Database allows patient follow-up despite a change in the treating facility, unlike records from individual institutions where patients may be lost to follow-up. Diagnosis and drug records are standardized and mapped to International Classification of Diseases, 10^th^ Edition (ICD-10) codes and Anatomical Therapeutic Chemical (ATC) codes, respectively. Data collected between April 1^st^, 2009 and March 31^st^, 2020 were analyzed to capture the prescription trends and pattern of all hypnotics, including MRA and ORA, which were introduced in Japan in 2010 and 2014, respectively.

In accordance with Japanese Ethical Guidelines for Medical and Biological Research Involving Human Subjects, ethics review and informed consent were not required for this study, which utilized commercially available de-identified secondary data provided from the JMDC Claims Database.

### Study population

We extracted data for patients aged ≥ 20 to < 75 years with a diagnosis of insomnia (ICD-10 code G470) who were prescribed ≥ 1 hypnotic, with continuous enrolment in the JMDC Claims Database for ≥ 12 months prior to the index date (the date of the first claim for a hypnotic in each analysis period). In the present study, we used the data for outpatients in the analyses. The 12-month period prior to the index date was defined as the baseline period. Patients with any of the following were excluded: ≥ 1 diagnosis of narcolepsy and/or cataplexy (G474) during the study period; hospitalization at the index date; missing data for the hypnotics prescription date during the study period; or use of hypnotics lacking prescription information during the study period. Patients who were prescribed hypnotics as *pro re nata* only at the index date and patients with overlapping prescriptions for hypnotics from ≥ 2 physicians at the index date were also excluded. Patient data were collected for each fiscal year (analysis period: April 1^st^–March 31^st^).

Eligible patients were classified as either new users or long-term users of hypnotics within each analysis period. New users were defined as individuals prescribed ≥ 1 hypnotic in any analysis period who had not been prescribed any hypnotics in their 12-month baseline period. Long-term users of hypnotics were defined as individuals who had a prescription for ≥ 1 hypnotic during each analysis period with a continuous prescription for hypnotics with the same mechanism of action (MOA) for ≥ 180 days prior to the index date. This definition of 180-day continuous prescription was chosen with reference to a prior systematic review in which a duration of 6 months or longer was the most common period for defining long-term BZD use [21] and other recent articles [22–25]. Individual patients could not be identified as both a new user and a long-term user in a single fiscal year.

### Hypnotics

Oral hypnotics indicated for insomnia in Japan at the time of the study are listed in Table 1; those prescribed for bedtime use only were analyzed. Drugs approved for the treatment of both anxiety and insomnia (e.g., etizolam, nitrazepam) were included if they were prescribed for bedtime administration. Because, in Japan, the prescription instructions including the administration timing are only recorded in the claims issued from pharmacies outside of medical institutions, any hypnotics prescribed during hospitalization or in examination rooms at outpatient visits could not be evaluated. Since the JMDC comprises inpatient and outpatient data, we only used outpatient data (as outpatients prescribed hypnotics with the instruction for bedtime use) in this study.

**Table 1:** Insomnia medications included in the analysis

**Generic name**	**Duration type**	**ATC code**	**Maximum number of days per prescription**
Benzodiazepines (BZD)			
Flurazepam	Long	N05CD01	30
Quazepam	Long	N05CD10	30
Haloxazolam	Long	-	30
Nitrazepam	Intermediate	N05CD02	90
Flunitrazepam	Intermediate	N05CD03	30
Estazolam	Intermediate	N05CD04	30
Nimetazepam	Intermediate	-	30
Lormetazepam	Short	N05CD06	30
Brotizolam	Short	N05CD09	30
Etizolam	Short	N05BA19	30
Rilmazafone hydrochloride	Short	-	Unrestricted
Triazolam	Ultrashort	N05CD05	30
Non-benzodiazepine hypnotics (z-drug)			
Zopiclone	Ultrashort	N05CF01	30
Zolpidem tartrate	Ultrashort	N05CF02	30
Eszopiclone	Ultrashort	N05CF04	Unrestricted
Melatonin receptor agonist (MRA)			
Ramelteon	-	N05CH02	Unrestricted
Orexin receptor antagonist (ORA)^a^			
Suvorexant	-	N05CM19	Unrestricted
Others			
Phenobarbital	-	N03AA02	30–90
Pentobarbital calcium	-	N05CA01	14
Amobarbital	-	N05CA02	14
Barbital	-	N05CA04	14
Chloral hydrate	-	N05CC01	Unrestricted
Bromovalerylurea	-	N05CM03	Unrestricted
Triclofos sodium	-	N05CM07	Unrestricted

*ATC* Anatomical Therapeutic Chemical

^a^ Lemborexant was not available in Japan in the study period

The hypnotics were divided into five classes according to their MOA: BZD, z-drug, MRA, ORA, and other hypnotics (Others). BZDs and z-drugs were further segmented by duration of action (long-acting type, intermediate-acting type, short-acting type, and ultrashort-acting type). Under the Japanese regulatory system, there is a 2-week prescription restriction that might limit long-term use for the first year on the market, and most drugs can be prescribed for long-term use after this 2-week restriction is removed in the second year or later. However, many hypnotics have a prescription limit (14–90 days). Table 1 also shows the maximum number of days permitted per prescription for hypnotics in Japan.

### Data analysis

The following demographic characteristics were analyzed descriptively for patients classified as new or long-term users of hypnotics in the last two analysis periods (fiscal years 2018–2019; i.e., April 1^st^, 2018 to March 31^st^, 2020): age, sex, medical specialty, clinical setting, and baseline psychiatric comorbidities. The medical specialty was defined based on the institution type as psychiatry (hospital or clinic with a primary specialty of psychiatry), general practice (GP; clinic with a primary specialty other than psychiatry), or others (hospital with a primary specialty other than psychiatry). The clinical setting was classified based on the number of beds as a clinic (0–19 beds) or hospital (≥ 20 beds). The following psychiatric comorbidities (ICD-10 codes) were considered: substance use disorders (F10–F19), schizophrenia spectrum disorders (F20–F29), bipolar disorders (F30, F31), depressive disorders (F32, F33), anxiety disorders (F40–F42), and neurocognitive disorders (F00, G30), and other psychiatric comorbidities (F00–F99, except for the psychiatric diseases designated above). As a neurocognitive disorder, we limited the analysis to patients with Alzheimer’s disease. The number of prescribed hypnotics (based on generic name) at the index date (1, 2, 3, or ≥ 4) was also analyzed.

All analyses were performed by prescription type at the index date (new or long-term users). The prescription trends were analyzed by calculating the yearly percentages of each hypnotic MOA class prescribed to new or long-term users, defined as the proportion of first hypnotic prescribed in each analysis period between April 1^st^, 2010 and March 31^st^, 2019. Prescription patterns of hypnotics were also assessed for the analysis periods 2018 and 2019 combined, to understand the recent treatment patterns of hypnotics prescribed to Japanese patients. New and long-term users were stratified by the MOA classes they were prescribed at the index date. In subgroup analyses, the data were segmented by age, sex, medical specialty, and psychiatric comorbidities (prescription pattern analysis only).

Data were analyzed descriptively. Continuous variables were summarized with the mean, standard deviation, 95% confidence interval (CI), median, minimum, and maximum. Categorical variables were summarized as the count (n) and proportion (%) with the 95% CI. All data analyses were performed using SAS® version 9.4 (SAS Institute, Cary, NC, USA).

## Results

### Patient disposition and characteristics

**Fig. 1:**
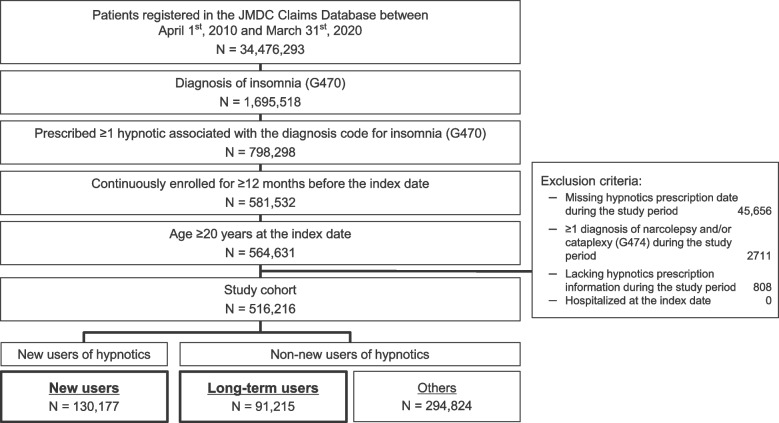
Patient flow diagram and identification of new and long-term users. The patient population in each box represents the cumulative number of patients in each analysis period

Of 34,476,293 cumulative patients registered in the JMDC Claims Database during the total study period (April 1^st^, 2010 to March 31^st^, 2020), 516,216 were extracted as the eligible patients who were prescribed ≥ 1 hypnotic associated with a diagnosis of insomnia (ICD-10 code G470) in outpatient settings (Fig. 1). These patients were subdivided into new users (130,177) and long-term users (91,215) of hypnotics. The numbers of new and long-term users in each analysis period (April 1^st^–March 31^st^) are listed in Table S1. The number of patients with insomnia increased with each year, mainly because of the increasing size of the database (Table S1). Tables S2 and S3 show the distribution of new and long-term users by age and sex for each analysis period.

The baseline characteristics of new users and long-term users of hypnotics in 2018–2019 are summarized in Table 2. The mean ± SD age of new and long-term users of hypnotics was 44.3 ± 13.0 years and 49.9 ± 11.3 years, respectively. There were slightly more males than females in both groups. The majority of new users were prescribed hypnotics by general practitioners at the index date, but this proportion was lower among long-term users. The most common baseline psychiatric comorbidities were depressive disorders in both new and long-term users, but the proportion of insomnia patients with depressive disorders was greater in long-term users (59.2%) than in new users (15.3%). From a treatment perspective, almost all new users were prescribed one hypnotic (96.3%), whereas 31.8% of long-term users were prescribed two or more hypnotics.

**Table 2:** Demographic characteristics of new users and long-term users of hypnotics in 2018 and 2019

	**New users of hypnotics**	**Long-term users of hypnotics**
N	55,263	42,444
Age		
Mean ± SD (95% CI)	44.3 ± 13.0 (44.2–44.4)	49.9 ± 11.3 (49.7–50.0)
Median (range)	45 (20–74)	50 (20–74)
Age group		
20–34	14,577 (26.4%) [26.0–26.7]	4487 (10.6%) [10.3–10.9]
35–49	19,779 (35.8%) [35.4–36.2]	15,208 (35.8%) [35.4–36.3]
50–64	17,757 (32.1%) [31.7–32.5]	18,794 (44.3%) [43.8–44.8]
65–74	3150 (5.7%) [5.5–5.9]	3955 (9.3%) [9.0–9.6]
Sex		
Male	29,892 (54.1%) [53.7–54.5]	22,900 (54.0%) [53.5–54.4]
Female	25,371 (45.9%) [45.5–46.3]	19,544 (46.0%) [45.6–46.5]
Medical specialty		
General practice	33,961 (61.5%) [61.0–61.9]	19,304 (45.5%) [45.0–46.0]
Psychiatry	16,513 (29.9%) [29.5–30.3]	20,658 (48.7%) [48.2–49.1]
Others	4789 (8.7%) [8.4–8.9]	2482 (5.8%) [5.6–6.1]
Clinical setting		
Clinic	46,258 (83.7%) [83.4–84.0]	32,524 (76.6%) [76.2–77.0]
Hospital	9005 (16.3%) [16.0–16.6]	9920 (23.4%) [23.0–23.8]
Baseline psychiatric comorbidity		
Depressive disorders	8482 (15.3%) [15.0–15.7]	25,147 (59.2%) [58.8–59.7]
Anxiety disorders	5925 (10.7%) [10.5–11.0]	10,147 (23.9%) [23.5–24.3]
Schizophrenia spectrum disorders	1966 (3.6%) [3.4–3.7]	11,501 (27.1%) [26.7–27.5]
Bipolar disorders	1304 (2.4%) [2.2–2.5]	7493 (17.7%) [17.3–18.0]
Substance use disorders	538 (1.0%) [0.9–1.1]	1219 (2.9%) [2.7–3.0]
Neurocognitive disorders	56 (0.1%) [0.1–0.1]	119 (0.3%) [0.2–0.3]
Others	8449 (15.3%) [15.0–15.6]	16,056 (37.8%) [37.4–38.3]
Number of hypnotics		
1	53,242 (96.3%) [96.2–96.5]	28,955 (68.2%) [67.8–68.7]
2	1982 (3.6%) [3.4–3.7]	12,389 (29.2%) [28.8–29.6]
3	38 (0.1%) [0.0–0.1]	990 (2.3%) [2.2–2.5]
≥ 4	< 5	110 (0.3%) [0.2–0.3]

Values are n (%) [95% CI] unless stated otherwise

As a neurocognitive disorder, we limited the analysis to patients with Alzheimer’s disease

*CI* confidence interval, *SD* standard deviation

### New users of hypnotics

#### Prescription trends (2010 to 2019)

**Fig. 2:**
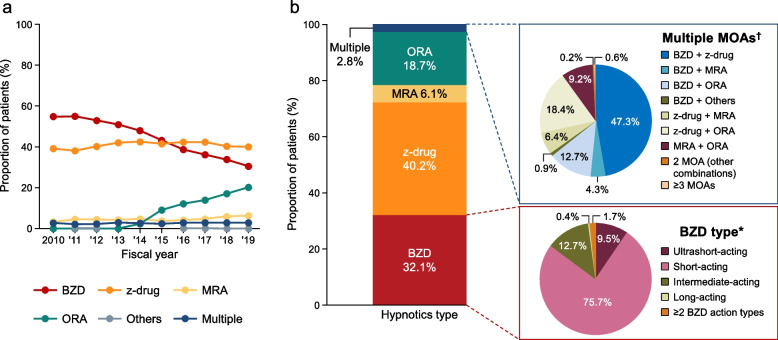
Trends in prescribed hypnotics (**a**) and distribution of baseline hypnotics (**b**) among new users. The trends in hypnotics prescribed between 2010 and 2019 (**a**) and the distribution of hypnotics prescribed in 2018–2019 (**b**) were assessed in new users of hypnotics. *The denominator was the number of patients prescribed BZDs (all BZDs were regarded as a single MOA regardless of the type). †The denominator was the number of patients prescribed multiple MOAs. *BZD* benzodiazepine, *MOA* mechanism of action, *MRA* melatonin receptor agonist, *ORA* orexin receptor antagonist, *z-drug* non-benzodiazepine

In 2010, almost all of the new users of hypnotics were prescribed GABA_A_-RAs (i.e., BZD [54.8%] or z-drugs [39.2%]); only ~ 6% of patients were prescribed other classes of hypnotics (MRA, multiple, or others) (Fig. 2a). The proportions of patients prescribed BZD declined over time from 54.8% in 2010 to 30.5% in 2019 whereas the proportion of patients prescribed z-drugs remained stable (~ 40%). The proportion of patients prescribed ORA increased from 2.3% in 2014 to 20.2% in 2019 while those for MRAs increased slightly, from 3.2% in 2010 to 6.3% in 2019. The proportion of new users prescribed multiple MOAs remained low (< 3%) in each year. The trends in hypnotics prescriptions were generally comparable among patients divided by age, sex, and medical specialty (Figures S1–S3). When analyzed by medical specialty (Figure S3), we found that BZDs were prescribed to fewer patients in GP (51.9% of patients in 2010 to 28.3% in 2019) than in psychiatry (60.3% of patients in 2010 to 35.4% in 2019). The proportions of patients prescribed z-drugs remained stable over time. From 2014 onwards, z-drugs were more frequently prescribed than BZDs in GP. Although prescriptions for BZDs in psychiatry steadily decreased, these drugs were more frequently prescribed than z-drugs throughout the analysis period. Prescriptions for ORA in psychiatry or in GP steadily increased to 21.5% and 19.9% of patients, respectively, in 2019. The proportion of patients prescribed an MRA in psychiatry increased slightly, from 1.9% in 2010 to 7.2% in 2019.

#### Treatment patterns (2018 and 2019)

In terms of treatments prescribed in the period 2018–2019, nearly all of the patients (97.2%) were prescribed a single MOA (Fig. 2b), with z-drugs being the most common types (40.2%), followed by BZD (32.1%), ORA (18.7%), and MRA (6.1%). Among BZD users, short-acting BZDs were the most common type (75.7%). Multiple MOAs were prescribed to 2.8% of patients, predominantly a BZD + z-drug (47.3%), followed by a z-drug + ORA (18.4%), and BZD + ORA (12.7%).

**Fig. 3:**
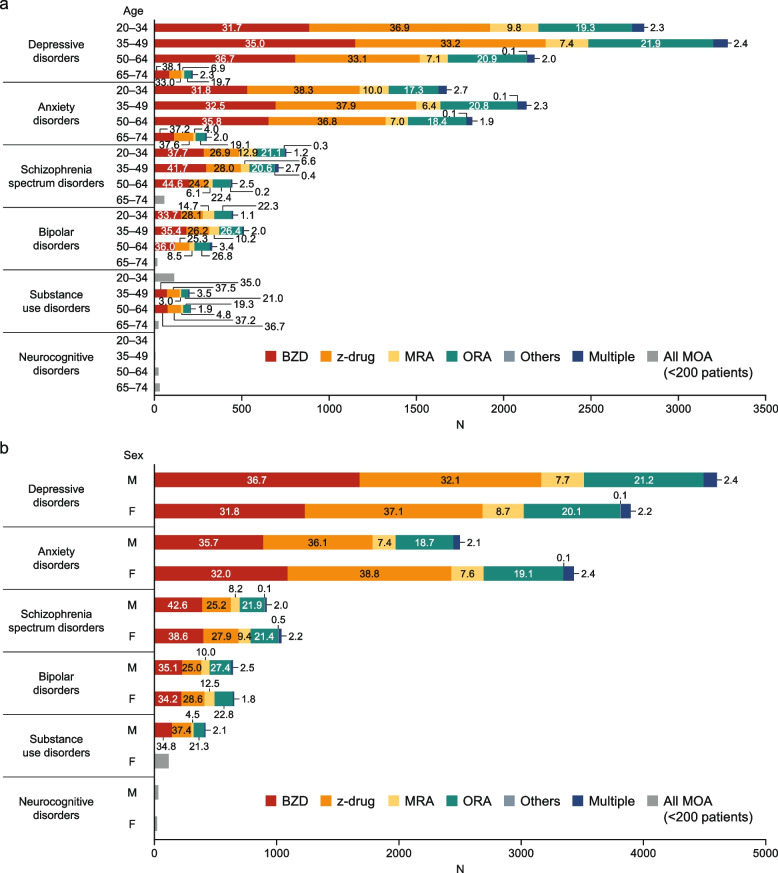
Distribution of hypnotics segmented by psychiatric comorbidities and age (**a**) and sex (**b**) among new users of hypnotics. The distributions of hypnotics prescribed to new users of hypnotics in 2018–2019 were segmented according to psychiatric comorbidities × age (**a**) and psychiatric comorbidities × sex (**b**). Values are % of patients. Neurocognitive disorders = Alzheimer’s disease. *BZD* benzodiazepine, *MOA* mechanism of action, *MRA* melatonin receptor agonist, *ORA* orexin receptor antagonist, *z-drug* non-benzodiazepine

The types of MOAs in new users were also broken down by age, sex, medical specialty, and psychiatric comorbidities (Figure S4). The patterns in hypnotics prescriptions were generally comparable in patients divided by age, sex, medical specialty, and psychiatric comorbidities. z-drugs and BZDs were the most common MOAs among all subgroups of patients, and BZDs were less frequently prescribed in GP than in psychiatry. Furthermore, when the distribution of MOAs was analyzed according to psychiatric comorbidities with stratification by age or sex, BZD prescriptions tended to be more frequent among older subgroups and male patients (Fig. 3). z-drugs were also frequently used among these subgroups.

### Long-term users of hypnotics

#### Prescription trends (2010 to 2019)

**Fig. 4:**
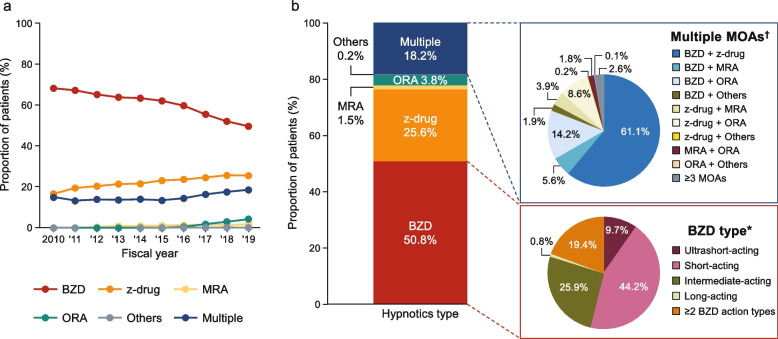
Trends in prescribed hypnotics (**a**) and distribution of baseline hypnotics (**b**) among long-term users. The trends in hypnotics prescribed between 2010 and 2019 (**a**) and the distribution of hypnotics prescribed in 2018–2019 (**b**) were assessed in long-term users of hypnotics. *The denominator was the number of patients prescribed BZDs (all BZDs were regarded as a single MOA regardless of the type). †The denominator was the number of patients prescribed multiple MOAs. *BZD* benzodiazepine, *MOA* mechanism of action, *MRA* melatonin receptor agonist, *ORA* orexin receptor antagonist, *z-drug* non-benzodiazepine

Among long-term users of hypnotics, the proportion of patients prescribed BZD declined over time, from 68.3% in 2010 to 49.7% in 2019 (Fig. 4a), while the use of z-drugs (from 16.7% to 25.6%), multiple MOAs (from 15.0% to 18.6%), and ORA (from 0% in 2010 to 4.3%) increased during this time. Unlike new users, a lower proportion of long-term users were prescribed ORA (excluding in combination with other drugs) in 2019.

The trends in hypnotics prescriptions were generally comparable among patients divided by age, sex, and medical specialty (Figures S5–S7). In each age group (Figure S5), the proportion of patients prescribed multiple MOAs tended to decrease and the proportion of patients prescribed z-drugs tended to increase with increasing age group. In males and females, the proportions of long-term users prescribed z-drugs, multiple MOAs, and ORAs tended to increase over time in both sexes (Figure S6). Regarding medical specialty, BZDs were the mainstay drugs prescribed in psychiatry or in GP, although prescriptions for BZDs decreased over time (Figure S7). z-drugs were prescribed to a greater proportion of patients in GP than in psychiatry, whereas multiple MOAs were prescribed to a greater proportion of patients in psychiatry than in GP.

#### Treatment patterns (2018 and 2019)

Among long-term users of hypnotics in 2018 and 2019, approximately half (50.8%) were prescribed BZD and one-quarter (25.6%) were prescribed z-drugs (Fig. 4b). Among patients prescribed BZD, short-acting BZDs were prescribed to 44.2% and intermediate-acting BZD to 25.9%; 19.4% were prescribed ≥ 2 duration types of BZD. Multiple MOAs were prescribed to a higher proportion (18.2%) of long-term users than to new users (2.8%). Among long-term users of multiple MOAs, BZD + z-drug (61.1%) and BZD + ORA (14.2%) were the most common combinations of MOAs.

**Fig. 5:**
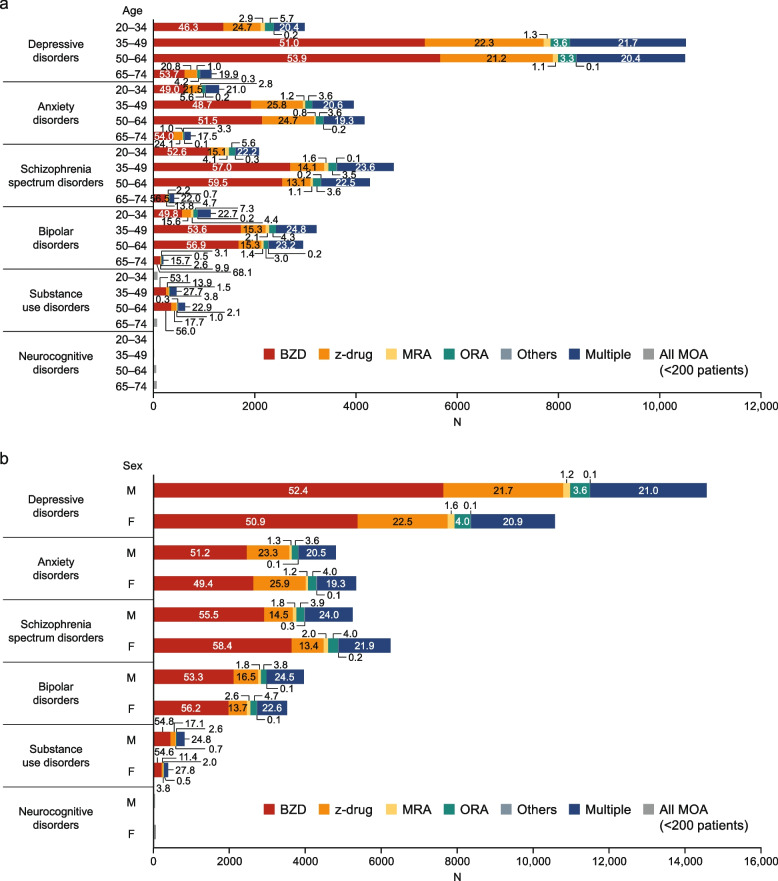
Distribution of hypnotics segmented by psychiatric comorbidities and age (**a**) and sex (**b**) among long-term users of hypnotics. The distributions of hypnotics prescribed to long-term users of hypnotics in 2018–2019 were segmented according to psychiatric comorbidities × age (**a**) and psychiatric comorbidities × sex (**b**). Values are % of patients. Neurocognitive disorders = Alzheimer’s disease. *BZD* benzodiazepine, *MOA* mechanism of action, *MRA* melatonin receptor agonist, *ORA* orexin receptor antagonist, *z-drug* non-benzodiazepine

The types of MOAs prescribed to long-term users were segmented by age, sex, medical specialty, and psychiatric comorbidities (Figure S8). The patterns in hypnotics prescriptions were generally comparable among patients divided by age, sex, medical specialty, and psychiatric comorbidities. BZD, z-drugs, and multiple MOAs were the most common types of therapies. In particular, z-drugs were prescribed to a lower percentage of patients and BZDs and multiple MOAs to greater percentages of patients in psychiatry than in GP. The distribution of MOAs among long-term users of hypnotics in 2018–2019 was also assessed according to psychiatric comorbidities stratified by age and sex (Fig. 5). The proportion of BZD prescriptions tended to increase with age.

## Discussion

We performed comprehensive analyses of the trends and current prescribing patterns of hypnotics for insomnia, including the use of newer MOAs such as MRA and ORA. Our results provide insight into the characteristics of patients prescribed hypnotics for the management of insomnia in the real-world setting in Japan. Recently, three studies that considered the introduction of ORA and MRA have been published in Japan [26–28], but the authors used different databases or did not examine how the introduction of newer classes of hypnotics influenced the prescribing patterns or trends in patients with insomnia. For the present study, we evaluated these outcomes in new users and in long-term users. The latter were defined as patients who received prescriptions for hypnotics of the same MOA for ≥ 180 days before the index date, and did not include patients who received continued prescriptions of hypnotics with different MOAs.

### Patient characteristics

One objective of this study was to evaluate the characteristics of new and long-term users of hypnotics in real-world clinical practice. Some key findings include the higher mean age of long-term users than new users (49.9 and 44.3 years, respectively). The mean age of new users in this study is similar to that reported in another study (42 years) in which the authors analyzed the same database, focusing on new BZD users between 2005 and 2014 [19]. On the other hand, the long-term users included a higher proportion of older patients, particularly in the age category 50–64 years (new users had a higher proportion in the category 20–34 years). Several previous studies demonstrated that age was associated with the long-term use of hypnotics [16, 18], and thus it is suggested that long-term use of hypnotics is more likely in older patients. Also, it has been reported that the prevalence of insomnia increases with age [16, 18, 19, 29, 30], partly as a result of the increased prevalence of comorbidities in older individuals. This evidence supports the present findings. However, because the database did not include any patients aged ≥ 75 years and comprised a small proportion of patients aged 65–74 years old, we could not reach clear conclusions regarding the patterns of hypnotics use among elderly patients.

We found that new users were predominantly treated in GP (clinics with a primary specialty other than psychiatry) whereas the long-term users were evenly split between GP and psychiatry (hospitals or clinics with a primary specialty of psychiatry). The higher proportion of long-term users treated in psychiatry may be explained by the high frequencies of psychiatric comorbidities, particularly depressive disorders, anxiety disorders, schizophrenia spectrum disorders, and bipolar disorders. The study included a very low proportion of patients with neurocognitive disorders (Alzheimer’s disease), which may be explained by the limited age range of patients registered in the JMDC Claims Database. The high proportions of patients with psychiatric disorders are unsurprising, and such patients frequently require long-term treatment with hypnotics, often with multiple MOAs or at high doses [31, 32].

Overall, these findings may suggest that (1) hypnotics are often started for insomnia patients at a relatively young age, but these patients have a lower rate of psychiatric comorbidities than older patients and are less likely to require long-term treatment; (2) insomnia patients with psychiatric comorbidities are more likely to visit psychiatrists for their insomnia therapy, and some elderly patients are prone to become long-term users of hypnotics; and (3) long-term pharmacological treatment may cause an increase in the number of hypnotics (multiple number of BZD or combination of multiple MOA hypnotics).

### Trends and patterns in new users of hypnotics

Insomnia is a challenging condition that is often undiagnosed, undertreated, or inappropriately treated. Hypnotics, particularly BZDs and z-drugs, have long been the mainstay pharmacological treatment for insomnia in Japan [7]. Among new users, nearly all (97.1%–97.9% in each analysis period) were prescribed a single MOA and, prior to ~ 2014, the majority were prescribed GABA_A_-RAs (i.e., BZD or z-drugs). This high frequency of GABA_A_-RA use is consistent with that reported in a study of 261,167 patients registered in the Medical Data Vision hospital-based administrative claims database for the period between 2012 and 2016 [26] in which BZDs and z-drugs were the first prescribed hypnotics in 59.7% and 36.8% of patients, respectively. For many years, GABA_A_-RAs have been the preferred insomnia medications due to limited treatment options [33–36]. The changes in the treatment landscape in Japan in the last decade have led to a reduction in the proportion of new users prescribed BZD. The proportion of patients prescribed z-drugs remained stable (~ 40%) and, since 2016, z-drugs have been the mainstay initial hypnotic for new users. This may reflect the understanding of the accumulating evidence of the risk–benefit balance of these drugs. Similar to us, Okui et al. reported that prescriptions of BZDs have steadily declined while prescriptions for z-drugs remained broadly unchanged among patients aged < 75 years at a university hospital in Japan [37], although the authors did not distinguish between new and long-term/chronic use. The prescription trends in our study were generally comparable among the four age groups, and were consistent with the trends for patients aged < 75 years reported by Okui et al. [37]. They also observed a decrease in prescriptions for BZDs in patients aged ≥ 75 years, while z-drug prescriptions increased through to ~ 2016 and declined thereafter. In our study, the use of ORA increased since the launch in 2014, reaching 20.2% of patients in 2019, while the use of MRA increased slightly (from 3.2% in 2010 to 6.3% in 2019). Based on these data, we surmise that physicians were more likely to consider ORA instead of BZD for new users of hypnotics.

The analyses of treatment of patients in 2018 and 2019 indicate that the majority of patients were prescribed a single MOA, and that GABA_A_-RAs were prescribed to ~ 70% of new users, with z-drugs being the mainstay treatment. ORA and MRA were prescribed to 18.7% and 6.1%, respectively. These patterns were generally consistent among the patient segments, although prescriptions for BZDs in patients with schizophrenia spectrum disorders, depressive disorders, and anxiety disorders tended to be more frequent among older patients and male patients compared with the other subgroup. Most BZD prescriptions were for short-acting formulations, similar to a study by Takano et al. [18] in which over half of new users of a BZD for hypnotic or anxiolytic purposes were prescribed short-acting (< 12 h) formulations. In the study by Inada et al., MRA was prescribed to 3.1% of patients [26], which is slightly lower than the proportion in our study based on more recent data. However, ORA was prescribed to only 0.4% of patients, which is much lower than the proportion in our study. One explanation is that the analysis period of that study was 2012–2016 and suvorexant was approved midway through the study (September 2014 and long-term use was permitted from December 2015 [38]). The study by Inada et al. [26] also involved patients initiating hypnotics at acute-care hospitals (inpatient or outpatient), whereas our study included only outpatients treated in community or hospital settings.

Multiple MOAs were prescribed to 2.8% of new users, despite Japanese guidelines [7], which recommend a single hypnotic as initial treatment. It is unclear why these new users received a prescription for hypnotics with multiple MOAs that did not conform to the guideline recommendations. It is possible that some physicians were writing prescriptions for the preferred combinations of hypnotics as initial hypnotics.

### Trends and patterns in long-term users of hypnotics

We also assessed the trends and patterns in prescription practices for long-term users of hypnotics. Similar to the analysis of new users, the prescriptions for BZDs to long-term users steadily declined over time, whereas the prescriptions for z-drugs steadily increased. The increase in prescriptions for ORA (from 0% to 4.3%) was lower than that in new users. There are several explanations for the steady decline in the use of BZDs among long-term users. A risk of BZD dependence among Japanese patients has been reported [39] and it is possible that the physicians became more aware of this risk following the alert issued by the Japanese Pharmaceuticals and Medical Devices Agency in 2017, which recommended limiting long-term/chronic use of BZDs [12]. Thus, some physicians may have switched patients from BZDs to other hypnotics to avoid these risks, and they were excluded from the eligible patients in the present study. Another factor that potentially influenced prescribing practices is the medical fee revision in 2018, which was partly aimed at reducing chronic BZD use [40, 41]. The introduction of MRA and ORA may also contribute to the decline in BZD prescriptions over time. However, it should be noted that approximately half (49.7%) were still continuously prescribed BZD for ≥ 180 days as of 2019. Several factors were reported to be associated with long-term use of hypnotics in prior studies. For example, Takano et al. demonstrated that patients with multiple factors, such as mood and neurotic disorders, patients with cancer, patients treated by a psychiatrist, patients who received multiple prescriptions, patients who were prescribed BZD formulations with a medium half-life, and elderly patients, were more likely to continue treatment with a BZD for > 8 months [18]. Similarly, Enomoto et al. reported that combined use of antidepressants, age, and mean dose were significantly associated with long-term use of hypnotics [16]. In addition, a study using Israel’s largest health record database demonstrated that one in five patients newly prescribed BZDs or z-drugs will continue using these drugs for up to 10 years [42]. We should also consider that some patients with insomnia were seeing the same psychiatrist for a long time. In such cases, both patients and physicians may be reluctant to switch from a BZD, with which they are familiar, to alternative hypnotics. Although the overall percentage of BZD prescriptions has steadily decreased, these factors may explain why some long-term users were still prescribed BZD, especially in the older age groups. However, while chronic use of BZDs needs to be reduced, it is also true that some patients with insomnia require long-term prescriptions for BZDs. In addition, it should be noted that there is limited information on the long-term effectiveness and tolerability of many hypnotics including MRA and ORA [43]. Therefore, physicians should take appropriate caution when prescribing hypnotics to patients over a long period of time.

Prescriptions for multiple MOAs increased slightly, from 15.0% to 18.6%. This slight increase may be correlated with the availability of ORA because 22.8% of multiple MOA users were prescribed BZD + ORA (14.2%) or z-drug + ORA (8.6%). Although a polypharmacy reduction policy, intended to reduce reimbursement and medical costs, was introduced in Japan in 2012 [40, 44], its impact could not be fully ascertained because only 2.6% of long-term users were prescribed ≥ 3 hypnotics and the trends in the distribution of hypnotics type among long-term users prescribed multiple MOAs were not assessed. There is a possibility that the physicians were lowering the doses of BZDs in combination with increased use of other hypnotics with different MOAs; however, this possibility could not be investigated in this study.

Although the proportion of long-term users prescribed ORA increased slightly over time, it remained low (3.8% in 2018 and 2019). In addition, a similar proportion (4.5%) of long-term users were prescribed two MOAs including ORA. Considering the definition of long-term use as continued prescription of the same MOA for ≥ 180 days in the present study, this finding may be supported by the recent findings obtained from Japanese post-marketing surveillance of suvorexant [45] and an analysis of prescribing patterns for hypnotic drugs in Japan using a hospital-based administrative claims database [26], in which the mean durations of ORA prescriptions were 62 days and 1.29 months, respectively. However, the proportion of patients continuing ORA therapy in the real-world clinical setting cannot be determined because patients who did not continue ORA for ≥ 180 days after transitioning to ORA or adding ORA to existing hypnotic treatment and patients who continued combination therapy including ORA for ≥ 180 days but changed the hypnotics other than ORA were excluded from the present study.

In the stratified analysis of long-term users in 2018 and 2019 of psychiatric comorbidities by age or sex, BZDs were the most commonly prescribed MOA, accounting for approximately 50% of patients. z-drugs and multiple MOAs were each prescribed to approximately 20% of patients. BZDs were also more frequently prescribed to older patients across all psychiatric comorbidities. ORA and MRA were prescribed to small proportions of patients in each segment. Slight differences among the segments may be related to differences in prescribing patterns in psychiatry than in GP, or the need to prescribe specific medications/combinations in consideration of the psychiatric comorbidity.

### Limitations

The database used in the present study enables us to provide valuable insight into the prescribing patterns and trends of hypnotics in new and long-term users because the database can track medical treatments despite a change in the treating facility, and its characteristics make it suitable for the investigation of chronic diseases. However, several limitations of this study warrant mention. First, the patients registered in the JMDC Claims Database are < 75 years old and primarily working-age individuals and their families enrolled in non-governmental healthcare insurance schemes. The number of patients aged ≥ 65 years is small and none were ≥ 75 years old. Therefore, there are limited data for elderly patients with insomnia. In addition, insomnia patients who cannot work are potentially excluded because patients who receive public funding are not included in the database. Second, only hypnotics prescribed for once-daily administration at bedtime were considered as eligible hypnotics for the analyses; hypnotics prescribed for administration at both daytime and bedtime were excluded. Third, the study excluded patients who received continued prescriptions of hypnotics with a change in the combination of MOAs, which could introduce selection bias. If many of the excluded patients continued hypnotics with varying combinations of MOAs, the trends might not be consistent with the results of the present study. Fourth, “psychiatry” was defined as a medical institution, which considered psychiatry as the primary department in the JMDC Claims Database. If the medical institution considered psychiatry as a non-primary department, the institution might be classified within the category “others.” Therefore, data for “psychiatry” might be underestimated. Finally, the data extracted from the JMDC Claims Database are designed primarily for insurance purposes and not for research; therefore, data might be contaminated by misclassification of ICD-10 codes or diagnosis for receipts to receive reimbursements. We extracted data for patients with psychiatric comorbidities based on ICD-10 codes, but these data should be carefully reviewed with reference to data on these patients in registry studies (e.g., [46]).

## Conclusion

In conclusion, the present study revealed distinct patterns and trends in the prescriptions of hypnotics between new users and long-term users among outpatients in Japan. Our findings have revealed the changes in prescribing practices for insomnia in Japan that coincide with the changes in the treatment landscape and healthcare policies. Although some trends, including reduced prescriptions for BZDs over time, were apparent for both new and long-term users, BZDs were more frequently prescribed to long-term users than new users. Further understanding of the treatment options for insomnia with accumulating evidence regarding the risk–benefit balance might be beneficial for physicians prescribing hypnotics in real-world settings.

## Supplementary Information

**Additional file 1:** **Table S1. **Profile of insomnia patients prescribed hypnotics in the JMDC Claims Database from 2010–2019 by fiscal year.** Table S2. **Proportions of new users of hypnotics by age and sex per analysis period.** Table S3. **Proportions of long-term users of hypnotics by age and sex per analysis period.** Figure S1. **Trends in prescriptions of hypnotics among new users of hypnotics by age.** Figure S2. **Trends in prescriptions of hypnotics among new users of hypnotics by sex.** Figure S3. **Trends in prescriptions of hypnotics among new users of hypnotics by medical specialty.** Figure S4. **Distribution of MOAs among new users by age (a), sex (b), psychiatric comorbidities (c), and medical specialty (d).** Figure S5. **Trends in prescriptions of hypnotics among long-term users of hypnotics by age.** Figure S6. **Trends in prescriptions of hypnotics among long-term users of hypnotics by sex.** Figure S7. **Trends in prescriptions of hypnotics among long-term users of hypnotics by medical specialty.** Figure S8. **Distribution of MOAs among long-term users by age (a), sex (b), psychiatric comorbidities (c), and medical specialty (d).

## Data Availability

The JMDC Claims Database analyzed in this study (analysis dataset) is not publicly accessible, and the data cannot be shared with external researchers according to the contract with JMDC Inc. Please contact JMDC Inc. (https://www.jmdc.co.jp) for inquiries about access to the dataset used in this study.

## Abbreviations

ATC: Anatomical Therapeutic Chemical
BZD: Benzodiazepine
CBTi: Cognitive behavioral therapy for insomnia
GABA_A_-RA: GABA_A_-receptor agonists
GP: General practice
ICD-10: International Classification of Diseases, 10^th^ Edition
MOA: Mechanism of action
MRA: Melatonin receptor agonist
ORA: Orexin receptor antagonist
z-drug: Non-benzodiazepine
