# Supplementary material for: Hypnotic prescription trends and patterns for the treatment of insomnia in Japan: analysis of a nationwide Japanese claims database
Source: BMC Psychiatry. 2023 Apr 20;23:278. doi: 10.1186/s12888-023-04683-2 (PMC10120113; doi:10.1186/s12888-023-04683-2)
Supplement: Supplementary file 1 — Additional file 1: Table S1. Profile of insomnia patients prescribed hypnotics in the JMDC Claims Database from 2010–2019 by fiscal year. Table S2. Proportions of new users of hypnotics by age and sex per analysis period. Table S3. Proportions of long-term users of hypnotics by age and sex per analysis period. Figure S1. Trends in prescriptions of hypnotics among new users of hypnotics by age. Figure S2. Trends in prescriptions of hypnotics among new users of hypnotics by sex. Figure S3. Trends in prescriptions of hypnotics among new users of hypnotics by medical specialty. Figure S4. Distribution of MOAs among new users by age (a), sex (b), psychiatric comorbidities (c), and medical specialty (d). Figure S5. Trends in prescriptions of hypnotics among long-term users of hypnotics by age. Figure S6. Trends in prescriptions of hypnotics among long-term users of hypnotics by sex. Figure S7. Trends in prescriptions of hypnotics among long-term users of hypnotics by medical specialty. Figure S8. Distribution of MOAs among long-term users by age (a), sex (b), psychiatric comorbidities (c), and medical specialty (d). [file 12888_2023_4683_MOESM1_ESM.pdf]

SUPPLEMENTAL INFORMATION

Hypnotic prescription trends and patterns for the treatment of insomnia in Japan: analysis of a nationwide Japanese claims database

Shoki Okuda, Zaina P. Qureshi, Yukiko Yanagida, Chie Ito, Yuji Homma, Shigeru Tokita

**Contents**	**Page**
**Table S1** Profile of insomnia patients prescribed hypnotics in the JMDC Claims Database from 2010–2019 by fiscal year	2
**Table S2** Proportions of new users of hypnotics by age and sex per analysis period	3
**Table S3** Proportions of long-term users of hypnotics by age and sex, per analysis period	4
**Figure S1** Trends in prescriptions of hypnotics among new users of hypnotics by age	5
**Figure S2** Trends in prescriptions of hypnotics among new users of hypnotics by sex	6
**Figure S3** Trends in prescriptions of hypnotics among new users of hypnotics by medical specialty	7
**Figure S4** Distribution of MOAs among new users by age (a), sex (b), psychiatric comorbidities (c), and medical specialty (d)	8
**Figure S5** Trends in prescriptions of hypnotics among long-term users of hypnotics by age	9
**Figure S6** Trends in prescriptions of hypnotics among long-term users of hypnotics by sex	10
**Figure S7** Trends in prescriptions of hypnotics among long-term users of hypnotics by medical specialty	11
**Figure S8** Distribution of MOAs among long-term users by age (a), sex (b), psychiatric comorbidities (c), and medical specialty (d)	12

Table S1 Profile of insomnia patients prescribed hypnotics in the JMDC Claims Database from 2010–2019 by fiscal year

Year	Subjects registered in JMDC Claims Database	Patients with diagnosis of insomnia	Patients with diagnosis of insomnia and prescribed ≥1 hypnotic	Study cohort: All users	Study cohort: New users	Study cohort: Long-term users
	N	N (%)	N (%)	N	N	N
2010	932,265	36,817 (3.9)	14,739 (1.6)	5521	1880	795
2011	1,109,462	56,359 (5.1)	22,982 (2.1)	9591	3164	1500
2012	1,567,889	74,576 (4.8)	31,658 (2.0)	13,645	4482	1980
2013	2,448,338	107,898 (4.4)	47,192 (1.9)	21,516	5990	3524
2014	2,613,490	115,905 (4.4)	51,903 (2.0)	36,954	9858	6282
2015	3,720,120	175,091 (4.7)	81,182 (2.2)	40,997	10,598	6970
2016	4,597,101	221,555 (4.8)	105,089 (2.3)	67,090	16,720	12,015
2017	5,389,907	269,157 (5.0)	130,800 (2.4)	88,858	22,222	15,705
2018	6,148,904	317,112 (5.2)	156,062 (2.5)	108,317	26,575	19,231
2019	5,948,817	321,048 (5.4)	156,691 (2.6)	123,727	28,688	23,213

Fiscal year = April 1st–March 31st

Table S2 Proportions of new users of hypnotics by age and sex per analysis period

**Analysis period**		**2010**	**2011**	**2012**	**2013**	**2014**	**2015**	**2016**	**2017**	**2018**	**2019**
N		1880	3164	4482	5990	9858	10,598	16,720	22,222	26,575	28,688
Age	20–34 yr	480 (25.5%)	787 (24.9%)	1115 (24.9%)	1418 (23.7%)	2377 (24.1%)	2530 (23.9%)	3861 (23.1%)	5070 (22.8%)	6719 (25.3%)	7858 (27.4%)
	35–49 yr	765 (40.7%)	1315 (41.6%)	1861 (41.5%)	2385 (39.8%)	3920 (39.8%)	4246 (40.1%)	6316 (37.8%)	8158 (36.7%)	9607 (36.2%)	10,172 (35.5%)
	50–64 yr	534 (28.4%)	905 (28.6%)	1247 (27.8%)	1765 (29.5%)	2934 (29.8%)	3155 (29.8%)	5471 (32.7%)	7423 (33.4%)	8612 (32.4%)	9145 (31.9%)
	65–74 yr	101 (5.4%)	157 (5.0%)	259 (5.8%)	422 (7.0%)	627 (6.4%)	667 (6.3%)	1072 (6.4%)	1571 (7.1%)	1637 (6.2%)	1513 (5.3%)
Sex	Male	1064 (56.6%)	1702 (53.8%)	2487 (55.5%)	3268 (54.6%)	5551 (56.3%)	5932 (56.0%)	9232 (55.2%)	12,207 (54.9%)	14,426 (54.3%)	15,466 (53.9%)
	Female	816 (43.4%)	1462 (46.2%)	1995 (44.5%)	2722 (45.4%)	4307 (43.7%)	4666 (44.0%)	7488 (44.8%)	10,015 (45.1%)	12,149 (45.7%)	13,222 (46.1%)

Table S3 Proportions of long-term users of hypnotics by age and sex per analysis period

Analysis period		2010	2011	2012	2013	2014	2015	2016	2017	2018	2019
N		795	1500	1980	3524	6282	6970	12,015	15,705	19,231	23,213
Age	20–34 yr	89 (11.2%)	207 (13.8%)	243 (12.3%)	399 (11.3%)	674 (10.7%)	786 (11.3%)	1283 (10.7%)	1580 (10.1%)	1976 (10.3%)	2511 (10.8%)
	35–49 yr	346 (43.5%)	609 (40.6%)	824 (41.6%)	1384 (39.3%)	2452 (39.0%)	2655 (38.1%)	4271 (35.5%)	5726 (36.5%)	6937 (36.1%)	8271 (35.6%)
	50–64 yr	278 (35.0%)	542 (36.1%)	717 (36.2%)	1246 (35.4%)	2369 (37.7%)	2737 (39.3%)	5206 (43.3%)	6772 (43.1%)	8453 (44.0%)	10,341 (44.5%)
	65–74 yr	82 (10.3%)	142 (9.5%)	196 (9.9%)	495 (14.0%)	787 (12.5%)	792 (11.4%)	1255 (10.4%)	1627 (10.4%)	1865 (9.7%)	2090 (9.0%)
Sex	Male	425 (53.5%)	822 (54.8%)	1093 (55.2%)	1901 (53.9%)	3332 (53.0%)	3775 (54.2%)	6584 (54.8%)	8634 (55.0%)	10,443 (54.3%)	12,457 (53.7%)
	Female	370 (46.5%)	678 (45.2%)	887 (44.8%)	1623 (46.1%)	2950 (47.0%)	3195 (45.8%)	5431 (45.2%)	7071 (45.0%)	8788 (45.7%)	10,756 (46.3%)

Figure S1 Trends in prescriptions of hypnotics among new users of hypnotics by age

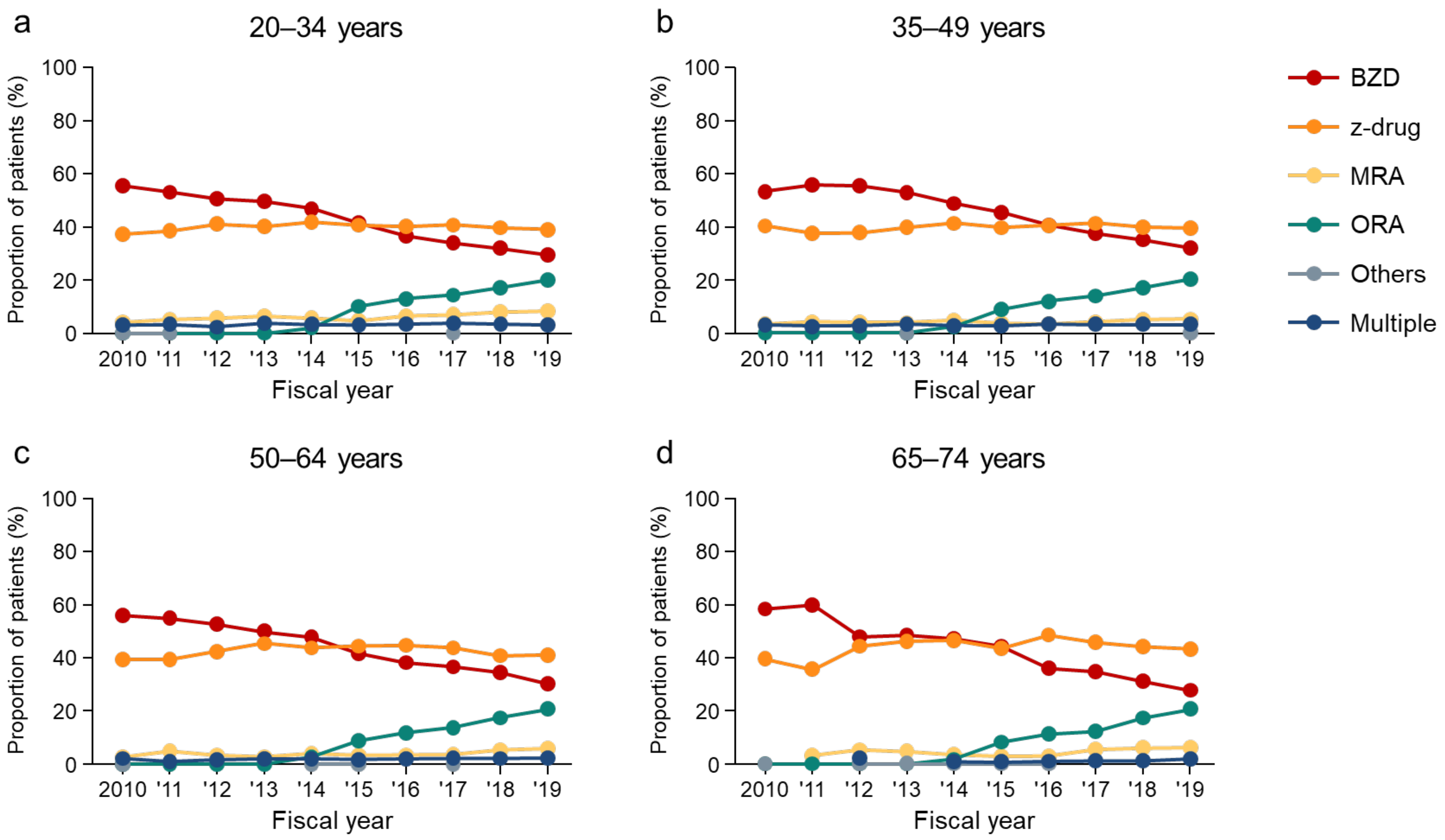

BZD benzodiazepine, *MRA* melatonin receptor agonist, *ORA* orexin receptor antagonist, *z-drug* non-benzodiazepine

Figure S2 Trends in prescriptions of hypnotics among new users of hypnotics by sex

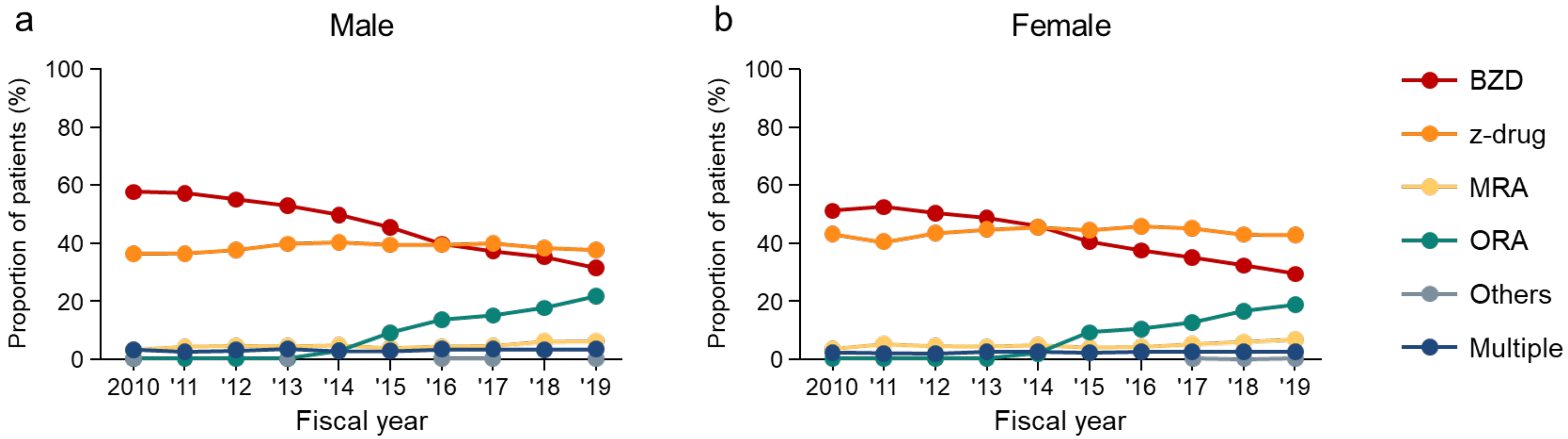

BZD benzodiazepine, *MRA* melatonin receptor agonist, *ORA* orexin receptor antagonist, *z-drug* non-benzodiazepine

Figure S3 Trends in prescriptions of hypnotics among new users of hypnotics by medical specialty

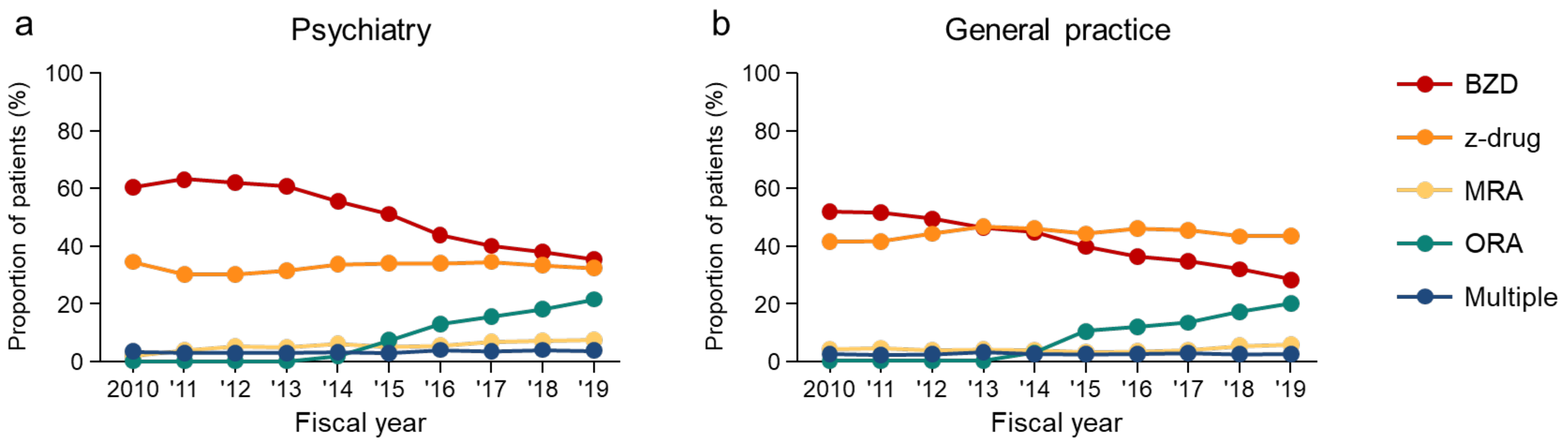

BZD benzodiazepine, *MRA* melatonin receptor agonist, *ORA* orexin receptor antagonist, *z-drug* non-benzodiazepine

Figure S4 Distribution of MOAs among new users by age (a), sex (b), psychiatric comorbidities (c), and medical specialty (d)

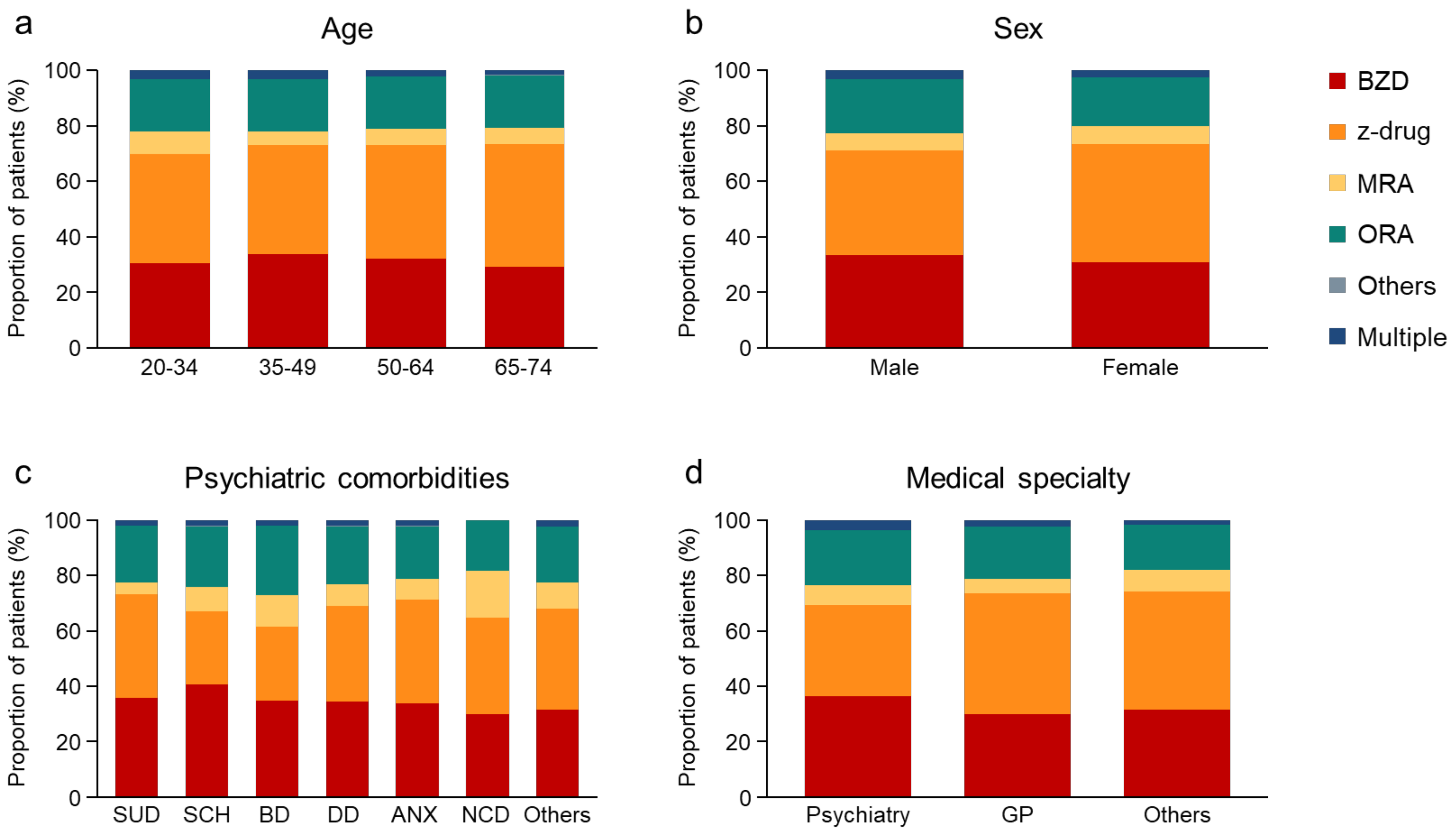

ANX anxiety disorders, *BD* bipolar disorders, *BZD* benzodiazepine, *DD* depressive disorders, *GP* general practice, *MOA*, mechanisms of action, *MRA* melatonin receptor agonist, *NCD* neurocognitive disorders (Alzheimer's disease), *ORA* orexin receptor antagonist, *SCH* schizophrenia spectrum disorders, *SUD* substance use disorders, *z-drug* non-benzodiazepine

Figure S5 Trends in prescriptions of hypnotics among long-term users of hypnotics by age

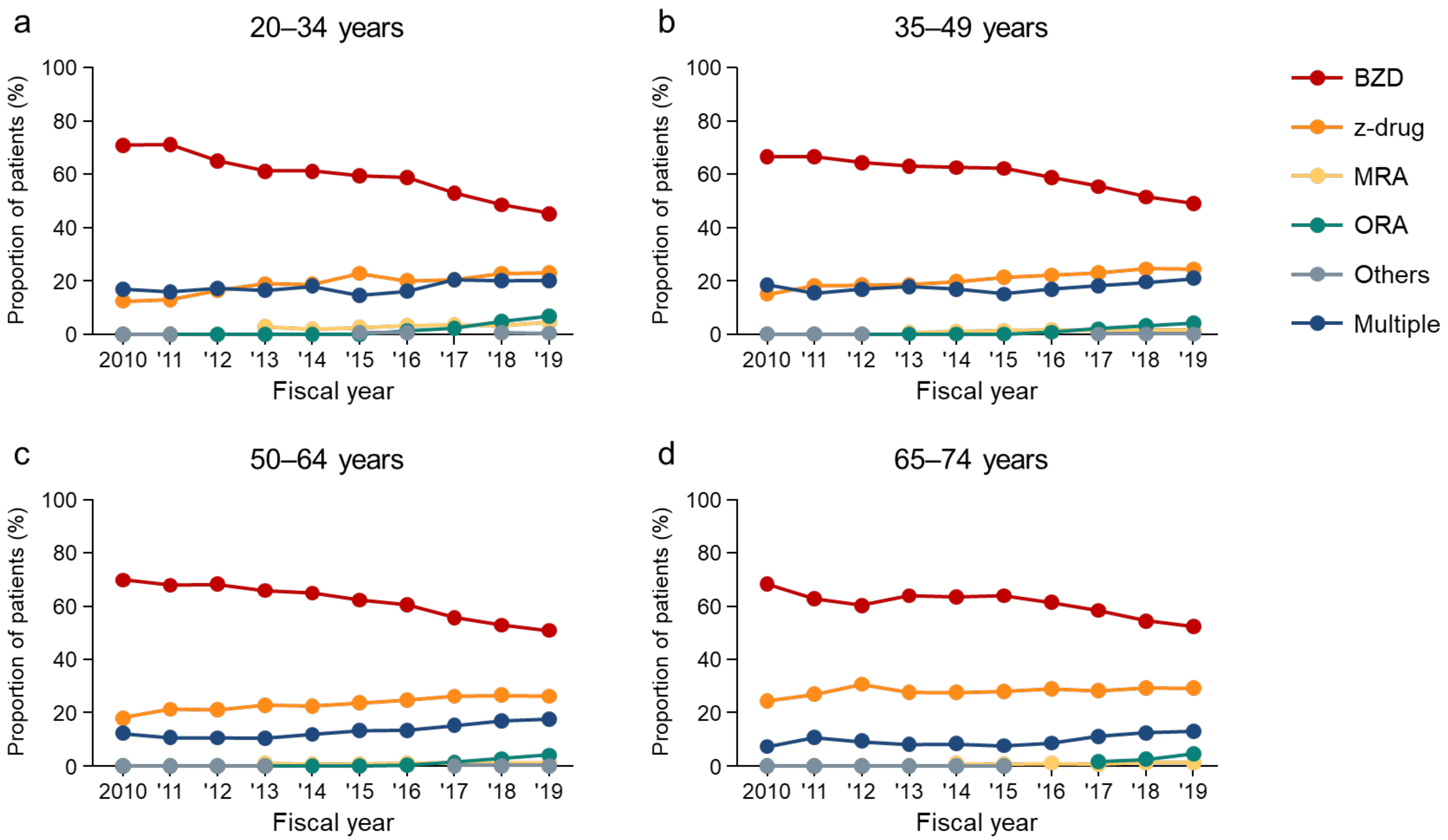

BZD benzodiazepine, *MRA* melatonin receptor agonist, *ORA* orexin receptor antagonist, *z-drug* non-benzodiazepine

Figure S6 Trends in prescriptions of hypnotics among long-term users of hypnotics by sex

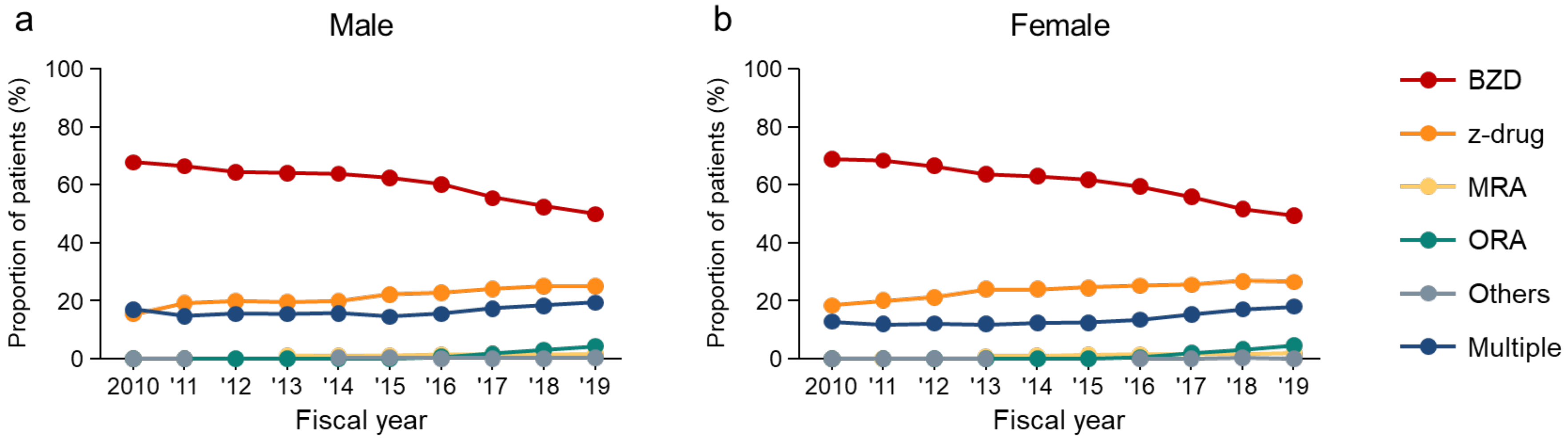

BZD benzodiazepine, *MRA* melatonin receptor agonist, *ORA* orexin receptor antagonist, *z-drug* non-benzodiazepine

Figure S7 Trends in prescriptions of hypnotics among long-term users of hypnotics by medical specialty

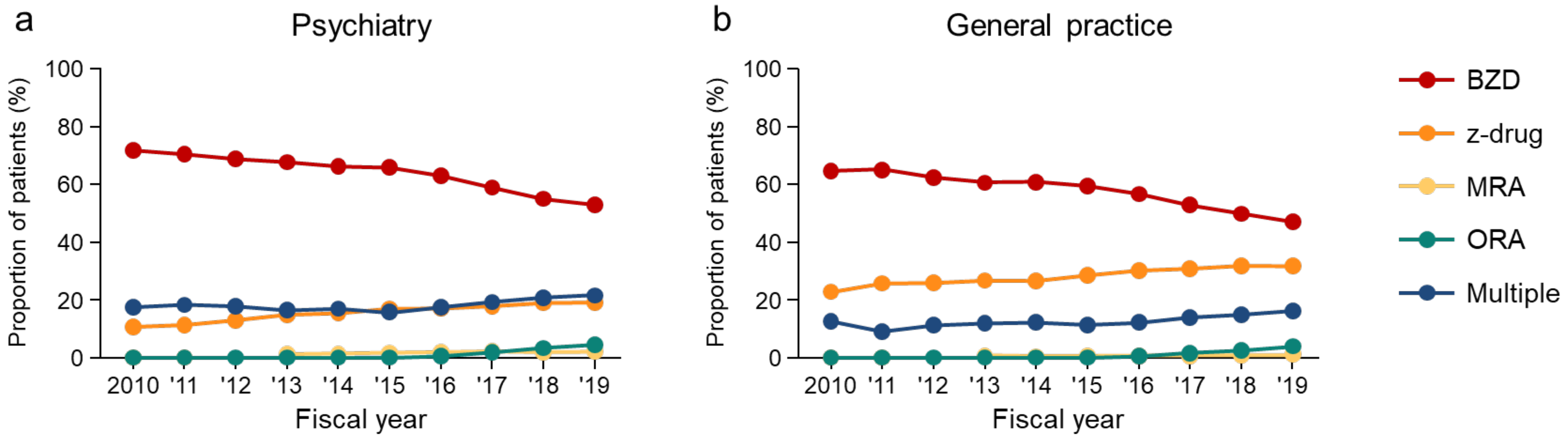

BZD benzodiazepine, *MRA* melatonin receptor agonist, *ORA* orexin receptor antagonist, *z-drug* non-benzodiazepine

Figure S8 Distribution of MOAs among long-term users by age (a), sex (b), psychiatric comorbidities (c), and medical specialty (d)

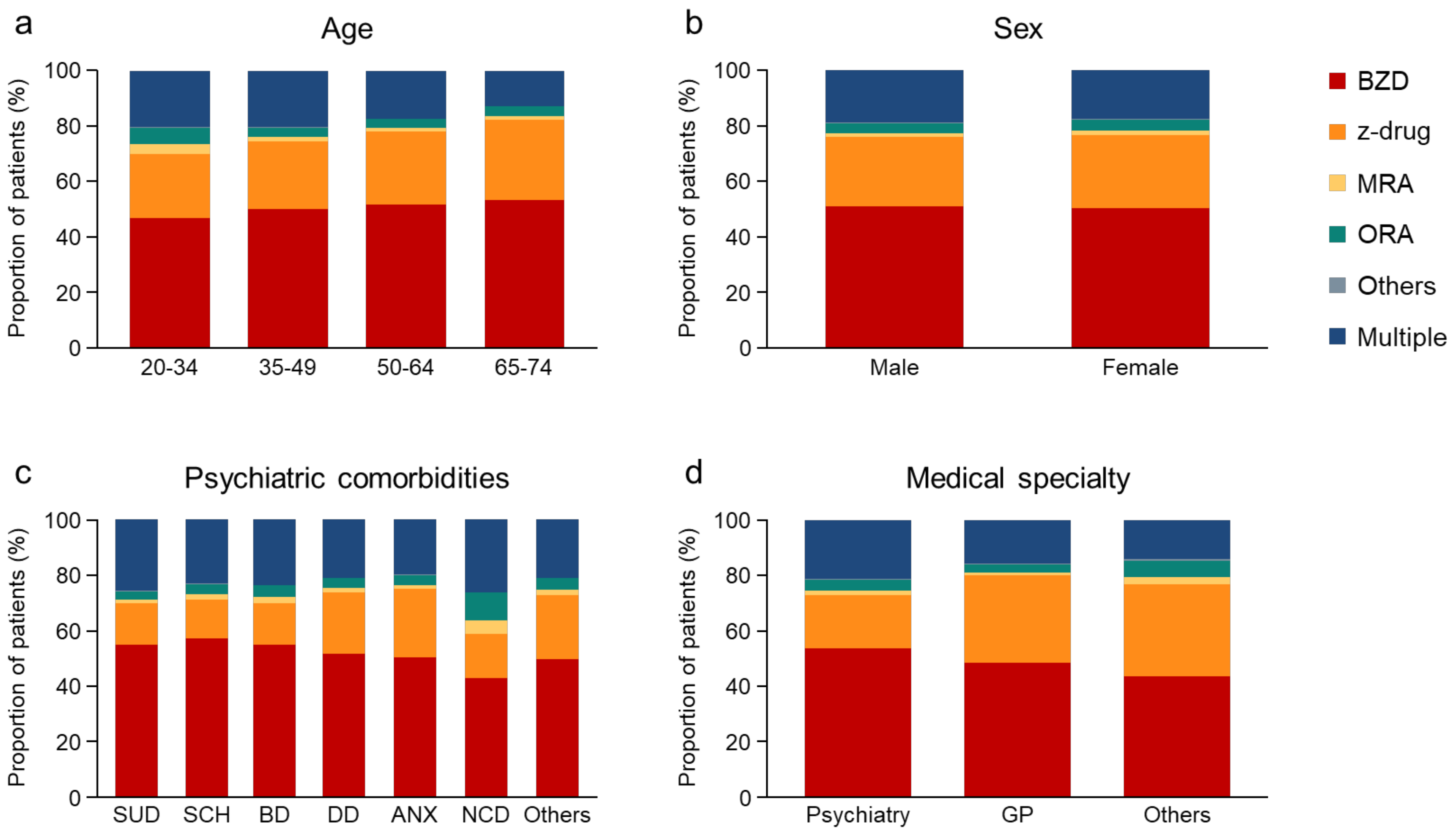

ANX anxiety disorders, *BD* bipolar disorders, *BZD* benzodiazepine, *DD* depressive disorders, *GP* general practice, *MOA*, mechanisms of action, *MRA* melatonin receptor agonist, *NCD* neurocognitive disorders (Alzheimer's disease), *ORA* orexin receptor antagonist, *SCH* schizophrenia spectrum disorders, *SUD* substance use disorders, *z-drug* non-benzodiazepine
